# Untangling the Manganese-α-Synuclein Web

**DOI:** 10.3389/fnins.2016.00364

**Published:** 2016-08-04

**Authors:** Tanara Vieira Peres, Nancy L. Parmalee, Ebany J. Martinez-Finley, Michael Aschner

**Affiliations:** ^1^Department of Molecular Pharmacology, Albert Einstein College of MedicineBronx, NY, USA; ^2^The Mind Research NetworkAlbuquerque, NM, USA

**Keywords:** manganese, neurotoxicity, alpha-synuclien, Parkinson disease, protein aggregation

## Abstract

Neurodegenerative diseases affect a significant portion of the aging population. Several lines of evidence suggest a positive association between environmental exposures, which are common and cumulative in a lifetime, and development of neurodegenerative diseases. Environmental or occupational exposure to manganese (Mn) has been implicated in neurodegeneration due to its ability to induce mitochondrial dysfunction, oxidative stress, and α-synuclein (α-Syn) aggregation. The role of the α-Syn protein vis-a-vis Mn is controversial, as it seemingly plays a duplicitous role in neuroprotection and neurodegeneration. α-Syn has low affinity for Mn, however an indirect interaction cannot be ruled out. In this review we will examine the current knowledge surrounding the interaction of α-Syn and Mn in neurodegenerative process.

## Manganese metabolism and neurotoxic aspects

**Figure 1:**
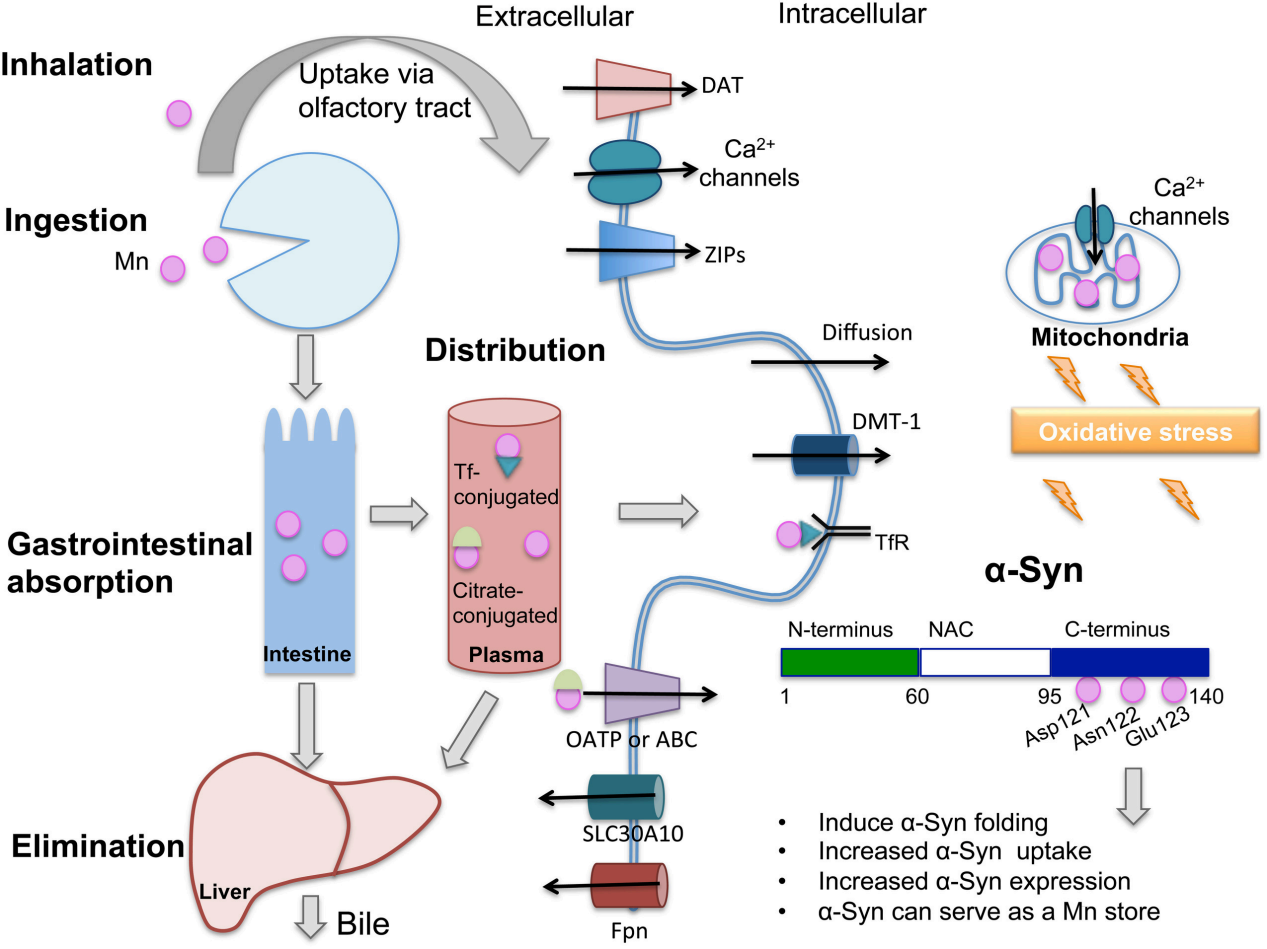
**Only 3–5% of ingested manganese (Mn) is absorbed through the intestinal epithelium and the majority is excreted via bile**. In plasma, Mn is found conjugated with transferrin (Tf), citrate or albumin (not shown), and distributed to various tissues. Mn can cross the blood-brain barrier (BBB) and access neurons and glia. Shown are proteins that participate in Mn transport across the plasma membrane: When conjugated with Tf and recognized by the transferrin receptor (TfR), Mn is endocytosed and can access the cytoplasm through divalent metal transporter-1 (DMT-1). Mn conjugated to citrate may be carried by members of the organic anion transporter polypeptide (Oatp) or ATP- binding cassette (ABC) superfamilies. Mn can also diffuse across the plasma membrane. Because they are both divalent, Mn can cross calcium (Ca^2+^) channels, which are also present in mitochondria. Excess Mn in mitochondria may induce oxidative stress. DMT-1 is the main transporter for Mn and other divalent ions. Mn uptake also occurs through the dopamine transporter (DAT). SLC30A10 is a newly characterized Mn exporter and ferroportin (FPN) has also been described to export Mn to the extracellular space. Mn binds to Asp-121, Asn-122, and Glu-123 residues of α-Synuclein (α-Syn), located in the C-terminus domain, a region with binding sites for various metals. This interaction has been shown to induce different consequences which are cited in the figure. Oxidative stress has been proposed to contribute to α-Syn oligomerization following exposure to Mn. DAT, dopamine transporter; DMT-1, divalent metal tranporter-1; ZIPs, zinc transporters that also show affinity for Mn; Tf, transferrin; TfR, transferrin receptor; NAC, Non-Aβ component.

Manganese (Mn) plays a necessary role in brain physiology and homeostasis. Although it is less abundant that other essential metals like iron and copper, it is a metalloenzyme and enzyme activator, playing crucial roles in cell homeostasis. Mn acts as a cofactor in pyruvate carboxylase (Scrutton et al., 1966), serine/threonine glutamine synthase, arginase, and manganese superoxide dismutase (MnSOD; Greger, 1999). The necessary dietary levels of Mn can easily be obtained by consumption of a variety of foods, such as green leafy vegetables, legumes, nuts, tea, chocolate, and fruits. Mn absorption is limited to 3–5% in adults (Davidsson et al., 1989a) and it occurs predominantly in the intestine. The distribution of Mn to various tissues occurs via plasma with Mn carrier proteins, such as transferrin (Tf; Davidsson et al., 1989b) and albumin or by conjugation with citrate. It has been demonstrated that the oxidative state of Mn in blood, influenced by plasma pH and ceruloplasmin activity(Jursa and Smith, 2009), can affect its binding to different carriers, with Mn in the trivalent state binding more avidly to Tf than Mn^2+^ (Davidsson et al., 1989b); however it is not clear whether distinct transition states influence Mn accumulation in brain. Mn reaches the brain by transport across the blood-brain barrier (BBB) via various mechanisms, including divalent metal transporter 1 (DMT1) transferrin receptor (TfR), calcium (Ca) channels, members of the organic anion transporter polypeptide (OATP), or ATP- binding cassette (ABC) superfamilies, in the case of Mn bound to citrate and diffusion. Uptake of Mn via the olfactory tract and trigeminal nerve also occurs. DMT1, transferrin receptor (TfR), zinc transporters (ZIP8 and ZIP14), the citrate and choline transporters, the dopamine transporter (DAT), and Ca^2+^ channels are responsible for Mn import to the cells in the central nervous system (CNS; Crossgrove et al., 2003; Crossgrove and Yokel, 2004, 2005; Roth, 2006). The essentiality of Mn means that the absorption and excretion of Mn must be tightly controlled to maintain stable tissue levels. The liver exerts this control. In the liver, Mn is removed from the blood, conjugated with bile and excreted into the intestine for elimination. Ferroportin (Fpn1) and SLC30A10 are primarily responsible for cellular efflux of Mn (Yokel et al., 2003; Quadri et al., 2012; Martinez-Finley et al., 2013; Chen et al., 2016). An overview of Mn metabolism and mechanisms of transport can be found in Figure 1.

Chronic overexposure to Mn can have a detrimental effect on the brain resulting in a disease state referred to as manganism. Manganism is characterized by behavioral changes, tremors, difficulty walking slow and clumsy movements, tremors, and facial muscle spasms. There are symptoms that precede advanced manganism, such as slowed hand movements, irritability, aggressiveness, and hallucinations. Like many other neurodegenerative diseases, patients may be asymptomatic for months or years after exposure (Huang et al., 1989; Crossgrove and Zheng, 2004; Cersosimo and Koller, 2006). It has also been suggested that Mn exposure could be an environmental factor for the development of other neurodegenerative diseases, such as Parkinson's disease (PD; Calne et al., 1994; Fukushima et al., 2010). At the cellular level, Mn toxicity is mediated by autoxidation of intracellular catecholamines, production of free radicals, reactive oxygen species, and toxic metabolites; depletion of cellular antioxidant defense mechanisms and alterations in mitochondrial function and ATP production. All of these factors may be related to deficits in dopaminergic (DAergic) neurotransmission and eventual degeneration of these neurons. Conversely, Mn deficiency could lead to impaired growth, skeletal abnormalities, ataxia, lipid, and carbohydrate metabolism defects and reproductive deficits (Aschner and Aschner, 2005). The primary source of Mn toxicity is occupational exposure. Of particular concern is inhalational exposure to welding fumes over a chronic period of time. Also concerning are exposures in those who work in the iron and steel, dry-cell battery, smelting, and ferromanganese industries (Josephs et al., 2005; Sriram et al., 2015). Significant levels of Mn can also be achieved through total parenteral nutrition and chronic liver failure (Nagatomo et al., 1999; Boggio Bertinet et al., 2000). Mn is also found as the fuel additive methylcyclopentadienyl Mn tricarbonyl (MMT), which is used as an anti-knock agent, increasing the levels of Mn in air (Gulson et al., 2006). Industrial activity may also elevate levels of Mn in air and water, contributing to widespread non-occupational Mn exposure (Carvalho et al., 2013; Oulhote et al., 2014; Bowler et al., 2015).

Although Mn is widely distributed in the environment, it is not clear if Mn exposure can induce early onset PD. Manganism and PD are two distinct syndromes, with overlapping dopaminergic symptoms. Mn affects the globus pallidus and striatum, while PD degeneration is concentrated to the substantia nigra pars compacta. Molecular characteristics of PD include: Impaired mitochondrial function, with consequent oxidative stress; abnormal vesicle processing; impaired proteassomal function; α-synuclein (α-Syn) aggregation; and Lewy bodies formation. Evidence of Mn interaction with PD-related gene products [parkin (PARK2), DJ-1 (PARK7), PINK1 (PTEN-induced putative kinase 1, PARK6), ATP13A2 (PARK9), and SLC30A10 as well as LRRK2 (leucine-rich repeat kinase 2, PARK8) and VPS35 (vacuolar protein sorting-associated protein 35, PARK17] has been reviewed (Roth, 2014) and it is likely that genetic predisposition combined with Mn exposure accelerates PD onset. In this review we will focus on Mn interaction with α-Syn. There are two aspects to be considered: (1) α-Syn influence over Mn induced toxicity (neuroprotection or aggravation of neurotoxicity (2) a possible contribution of Mn to α-Syn toxic actions (inducing fibril formation and aggregration).

## α-Synuclein and its roles in neurodegeneration

α-Syn is a small protein (~140 amino acids) that is expressed in the CNS and in red blood cells (Barbour et al., 2008). The synuclein family is made up of alpha (α)-, beta (β)-, and gamma (γ)- synucleins, which are predominantly localized to presynaptic neurons (α/β), and glia (γ) (Bendor et al., 2013). The γ form has also been shown in many cancers (Bendor et al., 2013). α-Syn, the focus of this review, is highly soluble, found in the cytosol and in presynaptic terminals near synaptic vesicles and can interact with lipid membranes. In the cytosol α-Syn is primarily unfolded but it can undergo many protein folding modifications including self-aggregation and formation of fibrils. α-Syn has a N-terminal, membrane binding structure, a central NAC domain, and a C-terminal domain (Figure 1). Vertebrates have seven 11 residue repeats in the N-terminus that are highly conserved, while worms, flies, and yeast do not contain α-Syn homologs (Bendor et al., 2013). The C-terminal end undergoes phosphorylation at multiple sites and is believed to be integral in the formation of fibrils but the mechanisms are unclear. Early intermediary oligomers of α-Syn appear to be the pathogenic species, rather than the mature fibrils (Xu et al., 2015). Of particular interest to this review, α-Syn exhibits an affinity for metals (Uversky et al., 2001; Binolfi et al., 2006) and it appears as though protein aggregation and cross-linking can be triggered by the presence of metals such as Aluminum (Al), Copper (Cu), Cadmium (Cd), Iron (Fe), Mn, and Zinc (Zn; Paik et al., 1999). It is unknown whether α-Syn can regulate intracellular levels of transition metals in a physiologically relevant way and how metals regulate posttranslational modifications of α-Syn.

The role of α-Syn in degeneration has been the focus of several lines of research, and the accumulation of misfolded α-Syn is used to define certain types of neurodegeneration (Bendor et al., 2013). α-Syn is of particular interest due to its role in Lewy-body (LB) formation in neurons of PD patients and accumulation in senile plaques in Alzheimer's disease (Ueda et al., 1993; Trojanowski et al., 1998; Goedert, 1999). Interestingly, monoclonal antibodies that are raised against Lewy bodies recognize α-Syn highlighting its role in PD (Giasson et al., 2000). PD has a complex etiology, with some forms sporadic and others due to proclivities in genetic makeup. α-Syn was the first gene identified as a genetic risk factor for autosomal-dominant PD (PARK1/SNCA; Gasser, 2009). Subsequent studies have further classified SNCA gene mutations to several point mutations duplications and triplications, A53T (Polymeropoulos et al., 1997; Li et al., 2001, 2004), A30P (Polymeropoulos et al., 1997; Li et al., 2001), E46K (Zarranz et al., 2004), H50Q (Appel-Cresswell et al., 2013), G51D (Lesage et al., 2013), and G209A (Papadimitriou et al., 1999). α-Syn aggregation in neurodegeneration is the best understood role for α-Syn, while the function of α-Syn under normal physiological conditions is more elusive. It can directly bind to the soluble N-ethylmaleimide-sensitive factor attachment protein receptor (SNARE)-protein synaptobrevin-2/vesicle-associated membrane protein (VAMP2) and promote SNARE complex assembly (Burre et al., 2010). α-Syn has also been shown to participate in dopamine biosynthesis and regulation (Perez et al., 2002). A relatively new theory is that α-Syn might spread in a prion-like process, self-propagating from the intestine, and in the CNS, progressing via transneuronal transport (Recasens and Dehay, 2014).

## Manganese interaction with α-synuclein

Metal dyshomeostasis is a feature present in many neurodegenerative disorders. Menke′s disease or Wilson's disease are linked to impaired Cu metabolism. Neurodegeneration with Brain Iron Accumulation refers to a group of several syndromes linked to impairment in Fe regulation. Furthermore, metals even at low concentrations can promote the oligomerization and aggregation of several proteins. Among these proteins are amyloid-beta (Aβ) that oligomerizes with Cu and Zn, amylin that oligomerizes in presence of Cu, tau protein that oligomerizes in presence of Al and Fe and α-Syn can oligomerize in presence of different metals including Al, Cu, Cd, and Fe (Carboni and Lingor, 2015). Mn has been shown to bind to Prion protein and potentially play a role in prion disease progression *in vivo* (Brazier et al., 2008; Choi et al., 2010). Mn can also bind to the N-terminal part of the Aβ (1-40) peptide, with a weak binding affinity in the milli- to micromolar range (Wallin et al., 2016).

α-Syn contains many divalent metal-binding sites in the region comprising residues 110–140, specifically Asp121, Asn122, and Glu123, additionally Cu can bind to His-50 (Rasia et al., 2005) and the Met-1 (Dudzik et al., 2013). Nuclear magnetic resonance (NMR) studies have shown that α-Syn has a poor affinity for Mn^2+^ in its C-terminal binding site, the range of affinity is in the 1 mM (Binolfi et al., 2006). Nevertheless, Mn^2+^ was shown to influence α-Syn folding in tyrosine fluorescence quenching assays but it failed to induce α-Syn fibril formation in aggregation assays with Thioflavin T fluorescence (Uversky et al., 2001). Furthermore, evidence points that Mn may regulate α-Syn homeostasis and transport through the blood-CSF barrier (BCSFB). Bates et al. (2015) used choroidal epithelial Z310 cells derived from the rodent choroid plexus and found that Mn exposure increased α-Syn uptake and intracellular accumulation without significantly changing mRNA expression. α-Syn expression was visualized using immunohistochemistry (Bates et al., 2015).

Further evidence for Mn and α-Syn interaction was found by Dučić et al. (2015), who evaluated the interplay between α-Syn and Mn in Mn-treated rat primary midbrain neurons overexpressing α-Syn using X-ray fluorescence imaging. They found that overexpression of α-Syn increased intracellular Mn levels of cells treated with 500 μM MnCl_2_ for 2 h and conversely decreased levels of other elements (Ca, Zn, K, P, and S). Cu, Fe, and Cl levels remained unaltered. The subcellular resolution revealed punctate Mn distribution, overlapping with DMT1 and MnSOD expression. This study suggests a role for α-Syn as an intracellular Mn store, however the authors do not show direct binding of Mn to α-Syn and the higher accumulation of Mn in these conditions could be due to decreased expression of Fpn1. Overexpression of α-Syn did not alter expression of the Mn import proteins DMT1 and voltage gated Ca channels (VGCC), but did attenuate Fpn1 and Mn release from Mn-treated neurons (Dučić et al., 2015). This finding indicates an indirect mechanism by which α-Syn regulates Mn accumulation.

The physiological concentration of Mn in the human brain is estimated to lie between 5.32 and 14.03 ng Mn/mg protein, equivalent to 20.0–52.8 μM Mn. The estimated pathophysiological threshold varies from 15.96 to 42.09 ng Mn/mg protein (60.1–158.4 μM Mn; Bowman and Aschner, 2014). Given that α-Syn possesses low affinity for Mn, it is likely that it will bind with Mn *in vivo* in cases of high Mn accumulation. It is possible, however, that Mn and α-Syn interact by indirect mechanisms. Mn may alter the accumulation of other metals, such as Cu and Fe (Fitsanakis et al., 2010; Angeli et al., 2014), which have shown high affinity for α-Syn. Alpha-Syn interacts with Cu in the μM range, with effects on its fibrillation and making it prone to metal-induced oxidation, which can also lead to protein aggregation (Binolfi et al., 2006). Thus, α-Syn influence on Mn neurotoxicity (either neuroprotection or aggravation of neurotoxicity), which will be discussed below, could be due to other actions of α-Syn and not necessarily its binding to Mn.

### α-Synuclein and neuroprotection against Mn-induced neurotoxicity

α-Syn was shown to be neuroprotective against Mn-induced neurotoxicity in a transgenic N27 dopaminergic neuronal cell line stably expressing human wild-type α-Syn at physiological levels (Harischandra et al., 2015). This cell line was compared to a control line expressing vector only. Neuroprotection, in the form of reducing cytochrome c release into the cytosol, was seen within a 24 h time period following exposure to 300 μM Mn. Therefore α-Syn appears to be protective against mitochondrial apoptosis early on. α-Syn also inhibited caspase-9 and -3 activation following Mn-exposure. The protective effect of α-Syn appeared to be independent of reactive oxygen species (ROS) inhibition, as ROS did not differ in any of the groups tested. At later time points, α-Syn and continued exposure to Mn promoted formation of α-Syn aggregates and it was no longer protective (Harischandra et al., 2015). These findings corroborate the notion of α-Syn as a Mn store, inducing protection. At later time points it is likely that increased accumulation of Mn induced α-Syn aggregration and toxicity.

Early onset PD-associated genes, parkin (PARK2) and PINK1 have been shown to work with α-Syn to regulate the mitochondrial stress response (Norris et al., 2015). In particular, parkin can inactivate α-Syn thereby suppressing mitochondrial fusion (Norris et al., 2015). *C. elegans* strains were used to conduct a comprehensive study of the interplay between wildtype α-Syn, Mn and mutated *pdr1* (parkin), *pink1*, and *djr1.1* (dj1). The study demonstrated rescue of oxidative stress, particularly reactive oxygen and nitrogen species, by α-Syn in the *pdr1* and *djr1.1* strains that were exposed to Mn compared to the deletion alone. α-Syn also decreased Mn accumulation in the *pdr1* and *djr1.1* mutants. They further probed *dat-1* (dopamine transporter) levels in these mutant strains and reported downregulation of *dat-1* in *djr1.1* mutants as compared to *pdr1* mutants, suggesting deficits in synaptic dopamine clearance in *djr1.1* mutants which was corroborated by increased neurodegeneration in α-Syn *djr1.1* mutants (Bornhorst et al., 2014).

It is important to note that wild type α-Syn was used in both of the studies showing neuroprotection against Mn exposure. Future studies should address the contribution of mutant α-Syn forms (i.e., A53T mutants) to neurodegeneration.

### α-Synuclein and aggravation of Mn-induced neurotoxicity

While the role of α-Syn in the absence of disease remains an open question, the participation of α-Syn in disease processes has been well established, with α-Syn fibrils being a major component of Lewy bodies. Upregulation of α-Syn has also been shown to induce neuronal cell death (Sung et al., 2001) and is accepted to be toxic to dopaminergic neurons (Li et al., 2010). The question of metal toxicity as a potential etiology for diseases of aging has been proposed based on known neurotoxic effects of metals combined with the reality that lifelong exposures to metals are usual and cumulative damage may appear in diseases of aging. There is evidence that Mn both alters the expression of α-Syn, leading to increased cellular levels of α-Syn in Mn treated cells (Cai et al., 2010; Li et al., 2010) and leads to aggregation of α-Syn, a hallmark of α-Syn in the disease state (Cai et al., 2010). Treatment of both SH-SY5Y human neuroblastoma cells, and PC12 cells with Mn resulted in toxicity and cell death with concomitant overexpression of α-Syn. Knockdown of α-Syn reduced the level of toxicity in response to Mn treatment, indicating that α-Syn participated in Mn toxicity.

Additional studies used human neuroblastoma (SK-N-MC) cells that stably expressed human dopamine transporter (DAT) and were transfected with human α-Syn and exposed for 24–72 h to 30–300 μM MnCl_2._ Mn treatment showed a synergistic exacerbation of cellular toxicity with α-Syn overexpression that was both concentration and time dependent suggesting that they work in concert with one another to produce neurodegeneration. The 30 μM group did not have significant differences but the cell loss was apparent in the 100 and 300 μM groups. No differences in DA transport were noted in this model (Pifl et al., 2004). These findings provide further evidence that one route of Mn toxicity may involve α-Syn overexpression and aggregation.

The mechanism of Mn-induced α-Syn toxicity remains under investigation. Mn toxicity is known to involve oxidative stress, but it is unknown whether this has a direct effect on α-Syn. To investigate the contribution of oxidative stress, rat brain slices were treated with Mn (II) chloride tetrahydrate, 0–400 μM for 24 h. Results showed concentration-dependent increases in ROS production, neuronal apoptosis and a decrease in SOD activity (Xu et al., 2013). Furthermore, α-Syn mRNA and protein expression was increased as were the appearance of α-Syn oligomers. The number of oligomers increased with increasing concentrations of Mn and were found to be mostly membrane bound. Protein carbonyl levels increased in a concentration-dependent fashion but were alleviated by pretreatment with GSH and aggravated by pretreatment with H_2_O_2_, as were α-Syn oligomers. This study highlighted the contribution of oxidative stress to α-Syn oligomerization following exposure to Mn (Xu et al., 2013).

Mn is known to exert toxic effects through more than one mechanism. Calpain 1 is a protease that has been shown to use α-Syn as a substrate. It has been hypothesized that α-Syn aggregation occurs through oligomerization of fragmented α-Syn. Calpain I was shown to play an important signaling role in Mn-induced α-Syn oligomerization (Xu et al., 2015). Xu and colleagues treated organotypic rat brain slices with 400 μM Mn^2+^ for 24 h and observed increases in the number of apoptotic cells (by up to 29.6%), lactate dehydrogenase release, intracellular calcium concentrations, calpain activity, mRNA and protein expression of calpain 1, and α-Syn. Calpain inhibitor II was used to pretreat cells prior to Mn exposure in an effort to explore the role of calpain in α-Syn oligomerization following Mn exposure (Xu et al., 2015). Pretreatment with calpain inhibitor II (4 μM) decreased the number of C- and N-terminal fragments of α-Syn and decreased overall oligomerization of α-Syn, suggesting that calpain-cleaved α-Syn fragments promote α-Syn oligomerization (Xu et al., 2015). This study provided further evidence that suggests that calpain I interacts with α-Syn in the cytoplasm of neuronal cells (Xu et al., 2015).

The effect of Mn and α-Syn on degeneration of dopaminergic cells was investigated using a mouse model. Mn interaction with human wild-type α-Syn was investigated using transgenic C57BL/6J mice expressing human α-Syn exposed to MnCl_2_ starting at 4 months of age. The study found that homovanillic acid (HVA)/dopamine (DA) ratios and aspartate levels were significantly increased in mice with human α-Syn compared to non-transgenic controls at 7 months of age. At that same time point, mice exposed to Mn had HVA/DA levels and aspartate levels that were significantly reduced in transgenic α-Syn mice but not in non-transgenic mice or mice with mutated α-Syn (A53T/A30P). This suggests that Mn interacted with human wild-type α-Syn influencing DA turnover even when there is no appreciable neurodegeneration (Peneder et al., 2011).

### Mn-induced α-synuclein aggregration

Several groups have investigated a mechanism for α-Syn aggregation in the presence of metals, including Mn. Given that aggregation of α-Syn in Lewy bodies is a hallmark of Parkinson's disease, insight into the interaction of α-Syn with metals could help to elucidate a mechanism for the formation of Lewy bodies lending insight into the etiology of Parkinson's disease. Binding features of several divalent metals, including Mn, were investigated using NMR spectroscopy to look at protein-metal interactions in an effort to identify possible binding sites for metals to α-Syn (Binolfi et al., 2006). This study identified a binding region for metals, including Mn, on the C-terminal portion of the α-Syn protein. However, this study showed a very low-affinity binding, in the millimolar range, suggesting that mechanisms other than direct binding may play a role in Mn-induced α-Syn aggregation. In a study by Uversky et al. (2001) it was shown that even low levels of metals were able to induce a conformational change in α-Syn, leading to the idea that a partially folded intermediate may act as a step in the process of aggregation, possibly through the process of ligand bridging by divalent metals, including Mn (Uversky et al., 2001).

α-Syn forms aggregates in the presence of Al, Cu, and Fe. To further investigate the mechanism by which Mn acts in the process of α-Syn oligomerization, Xu et al. (2014) explored the association between Mn-induced α-Syn oligomerization and S-nitrosylation of protein disulfide isomerase. They found that Mn induced nitrosative stress in the endoplasmic reticulum through activation of iNOS and S-nitrosylation of protein disulfide isomerase, a protein that is intimately linked to proper folding and maturation of native proteins. Cultured rat slices were treated with 0, 25, 100, or 400 μM Mn for 24 h. Results showed concentration-dependent increases in α-Syn oligomerization, apoptotic percentage of cells, lactate dehydrogenase release, NO production, inducible nitric oxide synthase activity and increases in mRNA and protein expression of iNOS and protein disulfide isomerase. Mn also increased α-Syn oligomerization and S-nitrosylated protein disulfide isomerase. Protein disulfide isomerase was S-nitrosylated at higher Mn levels, which significantly decreased its affinity for α-Syn. Pre-treatment of these slices with L-Canavanine (an iNOS inhibitor used to inhibit S-nitrosylation), administered prior to Mn, reduced Mn-induced α-Syn oligomerization, thereby highlighting the role of S-nitrosylation of protein disulfide isomerase in Mn-induction of α-Syn oligomerization (Xu et al., 2014).

In a multidisciplinary assessment of the effects of Mn exposure on 5–6 year old *Cynomolgus macaques* Verina et al. (2013) reported diffuse amyloid-β aggregation in the frontal cortex of Mn-exposed animals prompting them to assess α-Syn in their Mn-exposed primates. Mn was delivered via injection of 3.3–5.0, 5.0–6.7, 8.3–10.0 mg Mn/kg BW MnSO_4_. They reported increased α-Syn immunoreactivity in the frontal cortex gray matter and adjacent white matter of Mn-exposed primates. Multiple system atrophy, a type of progressive neurodegenerative disorder, was induced upon Mn exposure and appeared to have been driven by the aggregate formation of α-Syn, although the aggregation differed from animal to animal (Verina et al., 2013). An overview of publications regarding Mn interaction with α-Syn is available in Supplementary Table 1.

## Conclusion

The question of whether α-Syn is neurotoxic or neuroprotective and how Mn affects its role appears to be concentration and time dependent. Time frame for exposure and aggregation is a major consideration as α-Syn appears to be protective early on, often acting as a metal scavenger but later contributing to protein aggregation, neurodegeneration, and cell death. Mn can trigger misfolding and accumulation of α-Syn at certain concentrations. Some studies suggest that it can do this in the absence of increased mRNA expression, at least in the time frames tested. α-Syn's interaction with membranes, which extends to the endoplasmic reticulum and mitochondrial membranes as well as the plasma membrane, is likely the source of toxicity to the cells and eventual neurodegeneration and Mn appears to promote that process. However, it is not clear by the studies reviewed herein whether Mn interacts with α-Syn *in vivo* by a direct mechanism. It is suggested that Mn contributes to α-Syn aggregation, however current information support an indirect mechanism of Mn interaction with α-Syn, since it has been shown that Mn has low affinity for the protein and interaction only occurs in the millimolar range. Other metals, such as Cu and Fe, exhibit greater affinity for α-Syn and are known for inducing fibril formation. There is a complex interplay between metals, with each one influencing the homeostasis of the other. Therefore we cannot exclude heavy metal exposure as a molecular trigger to the development of PD and the elucidation of the mechanism by which these metals accumulate intracellularly and influence α-Syn aggregation could lead to another therapeutic target against PD. Additional studies that examine the effect of Mn vis-à-vis mutated forms of α-Syn in triggering neurodegeneration are also warranted to clarify a potential role of Mn in PD onset.

## Author contributions

TP, NP, EM, and MA conceived the article and wrote the manuscript. MA reviewed and edited the manuscript. All authors read and approved the manuscript.

## Funding

Generous funding from the National Institute of Environmental Health (R01ES010563 and R21ES025415-01A1).

### Conflict of interest statement

The authors declare that the research was conducted in the absence of any commercial or financial relationships that could be construed as a potential conflict of interest.
