# Supplementary material for: Untangling the Manganese-α-Synuclein Web
Source: Front Neurosci. 2016 Aug 4;10:364. doi: 10.3389/fnins.2016.00364 (PMC4972813; doi:10.3389/fnins.2016.00364)
Supplement: Supplementary file 1 [file Table1.DOCX]

**Table 1. Studies reporting on α-Syn and Manganese**

| Model | Treatment | Major Finding | Reference |
| --- | --- | --- | --- |
| Unlabeled and ^15^N-labeled wild-type α-Syn | MnCl_2_ 0.1-1.0 mM | - Mn binds to Asp-121, Asn-122, and Glu-123 - The range of affinity is 1 mM | ([Binolfi et al., 2006](#_ENREF_6)) |
| α-Syn solutions | MnCl_2_ 2 mM | - Mn influenced α-Syn folding - Mn did not induce α-Syn fibril formation | ([Uversky et al., 2001](#_ENREF_55)) |
| Choroidal epithelial Z310 cells derived from the rodent choroid plexus | MnCl_2_ 25-100 µM | - Mn exposure increased α-Syn uptake and intracellular accumulation without altering mRNA expression | ([Bates et al., 2015](#_ENREF_4)) |
| Rat primary midbrain neurons overexpressing α-Syn | MnCl_2_ /MnIII-pyrophosphate 500 µM/50 µM | - Overexpression of α-Syn increased intracellular Mn levels and decreased levels of other metals measured | ([Ducic et al., 2013](#_ENREF_24)) |
| Transgenic N27 dopaminergic neuronal cell line stably expressing human wild-type α-Syn | MnCl_2_ 300 µM | - α-Syn is protective against mitochondrial apoptosis early on. - Later time points, α-Syn and continued exposure to Mn promoted formation of α-Syn aggregates | ([Harischandra et al., 2015](#_ENREF_33)) |
| *Caenorhabditis elegans* (multiple strains) | MnCl_2_ (mM range-based on dose-response survival curve) | - α-Syn altered Mn accumulation in mutated *pdr1*, *djr1.1* worms | ([Bornhorst et al., 2014](#_ENREF_7)) |
| Human neuroblastoma (SK-N-MC) cells stably expressing human dopamine transporter (DAT) transfected with human α-Syn | MnCl_2_ 30-300 µM | - Mn treatment (concentration and time dependent) exacerbated α-Syn overexpression cellular toxicity | ([Pifl et al., 2004](#_ENREF_45)) |
| Organotypic slice cultures | MnCl_2_ 25-400 µM | - Dose-dependent increases in ROS, neuronal apoptosis  - Decreased SOD activity  - α-Syn mRNA and protein expression - increased α-Syn oligomers, mostly membrane bound | ([Xu et al., 2013](#_ENREF_60)) |
| Organotypic slice cultures | MnCl_2_ 25-400 µM | - Dose-dependent increases in α-Syn oligomerization, apoptotic percentage of cells, lactate dehydrogenase release, nitric oxide (NO) production, inducible nitric oxide synthase activity and increases in mRNA and protein expression of iNOS and protein disulfide isomerase - Mn also increased α-Syn oligomerization and S-nitrosylated protein disulfide isomerase | ([Xu et al., 2014](#_ENREF_58)) |
| Organotypic rat brain slices | MnCl_2_ 400 µM | - Increases in apoptotic cells, lactate dehydrogenase release, intracellular calcium concentration, calpain activity, α-Syn mRNA and protein levels | ([Xu et al., 2015](#_ENREF_59)) |
| Transgenic C57BL/6J mice expressing human α-Syn | 1% MnCl_2_ in food pellets | - α-Syn is important in Mn-induced DA turnover even when there is not appreciable neurodegeneration | ([Peneder et al., 2011](#_ENREF_43)) |
| *Cynomolgus macaques* | MnSO_4_ 3.3-5.0, 5.0-6.7, 8.3-10.0 mg Mn/kg BW | - Increased α-Syn immunoreactivity in the frontal cortex gray matter and adjacent white matter of Mn-exposed primates | ([Verina et al., 2013](#_ENREF_56)) |
